# Serum Thyrotropin and Phase of the Menstrual Cycle

**DOI:** 10.3389/fendo.2017.00250

**Published:** 2017-09-29

**Authors:** Salvatore Benvenga, Flavia Di Bari, Roberta Granese, Alessandro Antonelli

**Affiliations:** ^1^Department of Clinical and Experimental Medicine, University of Messina, Messina, Italy; ^2^Master Program on Childhood, Adolescent and Women’s Endocrine Health, University of Messina, Messina, Italy; ^3^Interdepartmental Program of Molecular & Clinical Endocrinology, and Women’s Endocrine Health, Azienda Ospedaliera Universitaria Policlinico “G. Martino”, Messina, Italy; ^4^Department of Human Pathology, University of Messina, Messina, Italy; ^5^Division of Obstetrics and Gynecology, University of Messina, Messina, Italy; ^6^Department of Clinical and Experimental Medicine, University of Pisa, Pisa, Italy

**Keywords:** thyrotropin, estradiol, menstrual cycle, refractory hypothyroidism, thyroid function tests

## Abstract

About one-fifth of patients treated with levothyroxine have serum thyrotropin (TSH) above target concentrations but, in approximately 15% of them, the cause of this TSH insufficient normalization remains unknown. We report the cases of two regularly menstruating women with known thyroid disease who had TSH levels consistently >3 mU/L (and sometimes above target levels) during mid-cycle, but consistently lower serum levels during the follicular and luteal phases of menstrual cycle. A major TSH release by the thyrotrophs in response to high circulating levels of estradiol (E2) at mid-cycle may increase levels of TSH compared to other phases of the cycle. The increased TSH can be misinterpreted as refractory hypothyroidism if the woman is under L-T4 replacement therapy or as subclinical hypothyroidism if the woman is not. Our findings might have important implications for diagnosis and management of thyroid disease, suggesting to request serum TSH measurements outside of the periovulatory days.

## Introduction

Approximately 20% of levothyroxine-treated patients have serum thyrotropin (TSH) above target level (1, 2). The ensuing biochemical and instrumental diagnostic work-up is multidisciplinary, can require hospitalization and be expensive. Yet, the cause of serum TSH insufficient normalization remains unknown in approximately 15% of patients (1).

A fortuitous circumstance, which occurred in the first patient reported here, led to the hypothesis that a robust TSH release by the thyrotrophs in response to high circulating levels of estradiol (E2) at mid-cycle may increase levels of TSH compared to other phases of the cycle. We report the two patients, because they could represent the classic tip of the iceberg.

## Background

The two women, aged 30 and 32 years, had primary hypothyroidism associated with Hashimoto’s thyroiditis, for which they were treated with approximately 1.5 μg/kg body weight/day levothyroxine by the endocrinologists who had observed the patients for the first time. Over the approximately 3 years of levothyroxine replacement therapy, both patients were always sampled between 8:00 and 8:30 a.m. in the same laboratory. The patients had no symptoms during L-T4 therapy; only patient A referred mild asthenia, but lower than in the pretherapy period. We excluded causes of elevated TSH in the face of levothyroxine therapy based on the extensive diagnostic work-up described previously (1). Particularly, non-compliance and pharmacological interference on the intestinal absorption of L-T4 were excluded by accurate medical history. Gastrointestinal diseases were excluded by the urea breath test for *H. pylori* infection, by measuring serum parietal cells antibodies (Ab), antitransglutaminase Ab and antiendomysium Ab. Furthermore, both women did not take any other drugs and they did not have gastrointestinal diseases.

Once we suspected the possibility of elevated serum TSH triggered by the elevation of serum E2 around midcycle, we also measured E2, prolactin (PRL) and gonadotropins [follicle-stimulating hormone (FSH) and luteinizing hormone (LH)].

The suspicion was fortuitous (see below, patient A) because, to obtain data on certain hormones assayed on days 1–7 of the follicular phase, we started recruiting regularly menstruating women with known thyroid disease.

**Figure 1:**
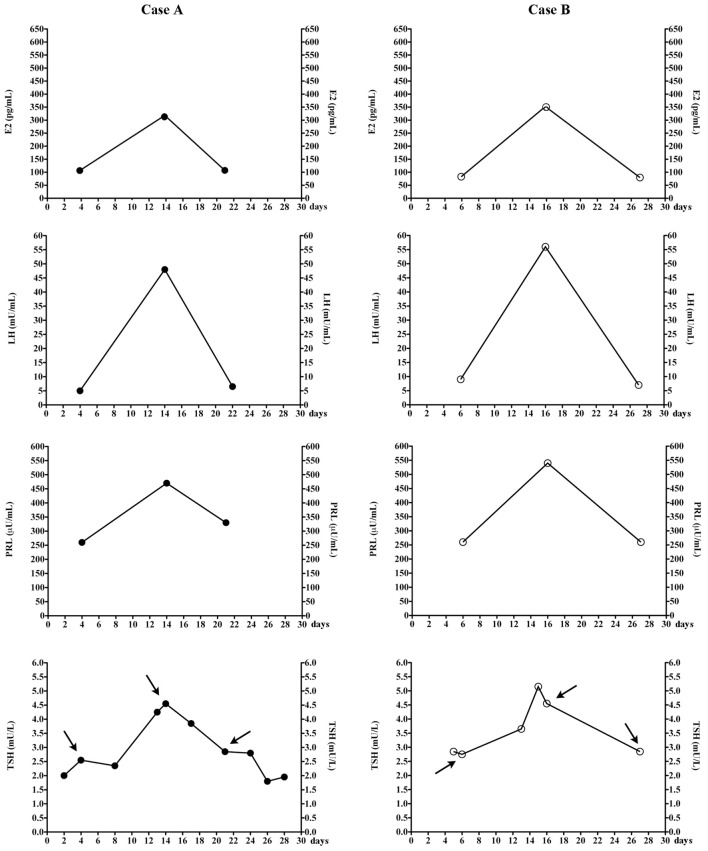
Changes of estradiol (E2), luteinizing hormone (LH), prolactin (PRL), and thyrotropin (TSH) in the two women, during the menstrual cycle. Arrows indicate TSH levels that were measured at the same time when the other hormones were also measured. The total number of menstrual cycles during which TSH levels were measured are nine for patients A and five for patients B. In our laboratory, follicular, midcycle, and luteal phase serum E2 ranges 23–139, 83–495, and 42–338 pg/mL (chemiluminescent assay by Beckman Coulter).

Patient A postponed sampling for the reproductive hormone assay because she wished to combine it with the TSH assay.*A posteriori*, this day of TSH sampling was mid-cycle, as she menstruated 13 days earlier and 15 days later. Serum TSH (4.6 mU/L) was the highest value recorded. Because of her regular menses (27- to 29-day duration), we easily matched her previous TSH assays with her menstrual cycle days (Figure 1). TSH levels and sometimes also FT4 levels were measured in several days of nine different menstrual cycles (Table 1). Serum E2, PRL, FSH and LH were also measured in the ovulatory and luteal phases of the 36th cycle and follicular phase of the 37th cycle (as indicated by the arrows in Figure 1). Serum TSH was <3.0 mU/L in both the luteal and follicular phase. Prior to levothyroxine therapy, TSH was 19.7 mU/L (mid-cycle), but it was 13.2 mU/L upon repeating the assay 19 days later (early follicular phase) to have confirmation of elevated TSH.

**Table 1:** Thyrotropin (TSH) and FT4 levels in the two women over the 37 or 34 menstrual cycles following initiation of L-T4 therapy, based on the day of the menstrual cycle.^a^

	Mentrual cycle since the beginning of L-T4 therapy	Corresponding day of the cycle	Corresponding TSH level, mU/L	Corresponding FT4 level, ng/dL
Case A	2nd	2nd	2.0	
	6th	28th	2.4	
	11th	17th	3.9	
	17th	8th	2.3	1.4 (0.7–1.8)
	21st	24th	2.8	
	25th	13th	4.2	
	31st	26th	1.75	1.45 (0.7–1.8)
	36th	14th	4.6	1.4 (0.7–1.8)
		21st	2.9	
	37th	4th	2.6	

Case B	3rd	5th	2.8	
	15th	13th	3.75	
	29th	15th	5.1	1.33 (0.9–1.6)
	33rd	16th	4.7	
		27th	2.8	
	34th	6th	2.6	

*^a^Prior to L-T4 therapy, in patient A, TSH was 19.7 mU/L in mid-cycle with FT4 0.63 ng/dL (0.7–1.48) but TSH was 13.2 mU/L in early follicular phase with FT4 0.65 ng/dL. In patient B, TSH was 27.4 mU/L in mid-cycle with FT4 0.78 ng/dL (0.9–1.6). In the laboratory, TSH normal range was 0.27 4.0 mU/L*.

Based on patient A, we wished to confirm these observations in another woman (patient B, Figure 1) in whom the diagnostic work-up for apparently undertreated hypothyroidism (1) had been inconclusive. Again, matching the days of blood sampling with the days of her regular menstrual cycle (Table 1), the two highest TSH values coincided with mid-cycle (Figure 1). Evaluated prospectively (as indicated by the arrows in Figure 1), the pattern held. The total number of menstrual cycles during which TSH levels were measured was five for patient B (Table 1).

In summary, except for the aforementioned peak values at mid-cycle, in the two women post-therapy serum TSH had been <3.0 mU/L when they were sampled between the 3rd–10th or the 20th–26th day of menstrual cycle, but it was greater when sampled in the periovulatory days.

## Discussion

To the best of our knowledge, we are unaware of cases similar to the two we are reporting here. In both patients, the highest TSH levels coincided with the highest levels of both estradiol (>300 pg/mL) and PRL, a well-known estrogen-upregulated hormone. Such estrogen dependency of serum TSH in the two women agrees with data of the literature (1, 3–8). These data are (i) the progressively increasing serum levels of TSH during gestation; (ii) the greater serum TSH levels in women with polycystic ovary syndrome or in women taking estroprogestins compared to control women, and in reproductive age women compared to men; (iii) the increased daily requirement of levothyroxine during endogenous or exogenous hyperestrogenism. Even in euthyroid postmenopausal women under estrogen therapy, serum TSH increased compared to pretherapy levels (5). In this study (5), in addition to 25 postmenopausal hypothyroid women (18/25 under levothyroxine replacement therapy), 11 euthyroid postmenopausal women were investigated. All women received conjugated estrogens in a daily oral dose of 0.625 mg for 48 weeks. Though in the euthyroid group pre-estrogen therapy serum TSH levels (1.3 ± 0.6 m/UL) were reported to be insignificantly changed over the 48 weeks of estrogen therapy, all post-therapy TSH levels, starting from the sixth week, were above baseline levels, approached or reached (48th week) 2.0 mU/L (5). In the hypothyroid group, serum TSH increased from 0.9 ± 1.1 to 3.2 ± 3.1 mU/L (*P* < 0.001), with levels greater than 7 mU/L in 7 of the 18 women (39%) under levothyroxine-replacement therapy (5).

Since it is known (9) that the thyrotrops have estrogen receptors, though not as abundant as other anterior pituitary cell types, and in view of literature on TSH responsiveness to E2 (10–12), we can elaborate a hypothesis for our findings in the two patients.

In ovariectomized rats, increasing serum estrogen levels within the physiological range increase both basal and TRH-stimulated TSH release as well as PRL release (10, 11). We hypothesize that a robust E2-driven TSH release at mid-cycle with consequent elevation of serum TSH may result from either a robust circulating E2 peak and associated hyper-response of the E2 receptors in the thyrotrops or increased sensitivity of E2 receptors to less robust circulating E2 levels. However, additional mechanism cannot be excluded. These mechanisms include the E2-driven (i) inhibition of the negative feedback that thyroid hormones exert on both basal and TRH-stimulated TSH release (12), (ii) induction of TRH receptors in the pituitary (13), and (iii) decreased TRH degradation (14).

## Concluding Remarks

In conclusion, a large prospective study is required to know the proportion of reproductive age women, under L-T4 replacement therapy, with elevation of serum TSH due to mid-cycle blood sampling. Once confirmed, our observations may have important implications for diagnosis and management of thyroid disease, and may call for standardization of serum TSH measurement outside of periovulatory days.

## Ethics Statement

This study was carried out with written informed consent from all subjects. All subjects gave written informed consent in accordance with the declaration of Helsinki. This study did not require submission to Ethical Committee.

## Author Contributions

SB collected data. All the authors contributed equally to the writing of the manuscript and approved the final version.

## Conflict of Interest Statement

The authors declare that the research was conducted in the absence of any commercial or financial relationships that could be construed as a potential conflict of interest.
